# Severe Malaria in an Adult Patient from Low-Endemic Area in Flores Island, East Nusa Tenggara

**DOI:** 10.1155/2023/1239318

**Published:** 2023-02-21

**Authors:** Maria Teressa, Asep Purnama, Joshua Henrina, Agustinus Wiraatmadja, Angela M. B. Boro, Candida I. L. Sam, Tersila A. D. Dedang, Alius Cahyadi

**Affiliations:** ^1^Department of Internal Medicine, T.C. Hillers General Public Hospital, Sikka Regency, East Nusa Tenggara 86113, Indonesia; ^2^Department of Neurology, T.C. Hillers General Public Hospital, Sikka Regency, East Nusa Tenggara 86113, Indonesia; ^3^Department of Internal Medicine, School of Medicine and Health Sciences, Atma Jaya Catholic University of Indonesia, Jakarta 14440, Indonesia

## Abstract

Malaria is an infection caused by protozoa of the genus *Plasmodium*, commonly found in tropical and subtropical regions worldwide. *Plasmodium falciparum* causes the most severe form of the disease and may progress to life-threatening manifestations. This case describes a 26-year-old man who suffered cerebral malaria with multiple organ dysfunction and successfully recovered despite poor initial prognosis. Negligent and late diagnosis of malaria leads to severe complications and a worse prognosis. This case emphasizes despite living in a low-endemic malaria area, physicians should remain meticulous and consider malaria as differential diagnosis even after initially presenting with nonspecific symptoms. Consequently, malarial screening should be performed to modify the risk of mortality. Furthermore, close monitoring and early administration of intravenous artesunate are also particularly critical.

## 1. Introduction

Malaria is a mosquito-borne disease commonly found in tropical and subtropical regions worldwide. *Plasmodium falciparum* causes the most severe form of the disease and contributes to the highest incidence of malaria infection. Infection from this parasite can be fatal without promptly recognizing the disease and its complications [1]. Cerebral malaria is one of the most advanced clinical courses of severe malaria, leading to life-threatening complications [2]. Herein, we present a case of a young adult who suffered severe cerebral malaria with multiple organ dysfunction and successfully recovered despite poor initial prognosis.

## 2. Case Presentation

**Figure 1:**
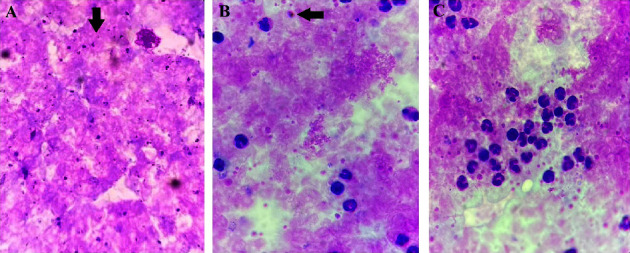
Peripheral blood smear of *Plasmodium falciparum* (arrows) legends. (a) Thick film showed asexual stage of *P. falciparum* on day 1. (b) The gametocytes of *P. falciparum* on day 3. (c) Asexual stage and gametocytes of *P. falciparum* were not found on day 4.

A 26-year-old male patient was referred to the General Public Hospital in Sikka Regency, East Nusa Tenggara, Indonesia, with presentations of loss of consciousness and jaundice. The patient initially developed fever, headache, and abdominal pain since 14 days before being admitted to our hospital. Several days before, he was treated for dyspepsia in another primary health center, which did not get better, and he was treated for the same diagnosis in a private clinic. As his symptoms were persistent, he was transferred to the regional public hospital in his area. At that moment, peripheral blood smear revealed *Plasmodium falciparum* and parasitemia with a density of 176,000 parasites/*μ*L (3.52%) (Figure 1). His past medical and travelling history was insignificant. He worked as a farmer and never used a mosquito repellent before. First dose of intravenous antimalarial artesunate (168 mg) was commenced, and repeated dose was followed at 12 hours afterwards. As he had an episode of seizure and acute kidney injury, after which his consciousness declined progressively, the patient was transferred to our hospital.

On clinical presentation, the patient was stupor, Glasgow coma scale 6, with profound jaundice, blood pressure was 142/75 mmHg, heart rate was 105 bpm, respiratory rate was 30 bpm, oxygen saturation was 96% using a nonrebreathing mask on 12 l/min oxygen, and the temperature was 38.5 C. Physical examination was significant for icteric sclera, copious hematemesis, hepatomegaly, splenomegaly, and black colored urine. Baseline and follow-up laboratory findings during hospitalization are shown in Table 1. Initial laboratory tests were notable for acute kidney injury (creatinine 12.8 mg/dL, urea 529 mg/dL, and blood urea nitrogen 246.87 mg/dL), elevated transaminases (aspartate aminotransferase 214 U/L and alanine aminotransferase 409 U/L), anemia (9.8 g/dL), and thrombocytopenia (61,000/*μ*L). He tested negative for HBsAg, negative for anti-HCV, and negative for anti-HIV. Chest X-ray demonstrated normal lung presentation.

The patient was diagnosed with severe malaria caused by *Plasmodium falciparum* with multiple organ dysfunction: acute symptomatic seizure, acute kidney injury, jaundice, anemia, and copious hematemesis. The patient underwent emergent hemodialysis and intravenous artesunate was given at 24 hours and continued daily. Other symptomatic and supportive treatments such as parenteral feeding, broad-spectrum antibiotic, and anticonvulsant were also given. On the second day, his clinical condition did not improve, although we observed initial downtrend in urea, serum creatinine, and liver enzymes levels. He remained feverish and agitated.

**Figure 2:**
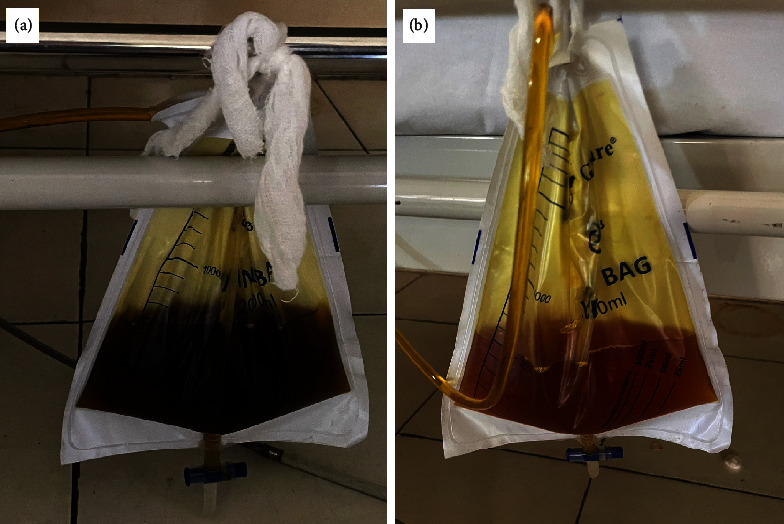
Urine collected on day 1 and day 3 since presentation legends. (a) Black-tea colored urine on day 1. (b) Yellowish-colored urine on day 3.

The second hemodialysis was performed in the following days, and he received 250 cc of packed red cell blood transfusion. A repeated malarial blood smear was negative for malarial parasite on the fourth day of hospitalization (Figure 1). Subsequently, on the seventh day, the patient's condition stabilized. He was alert, obeyed the command, and began enteral feeding. Intravenous artesunate was switched to oral dihydroartemisinin-piperaquine (160 mg/1,280 mg) and primaquine (15 mg). Physical examination revealed no icteric sclera and improved urine color (Figure 2).

Eventually, the patient underwent an uneventful fourteen days of care in our hospital. His hospitalization concluded with six doses of intravenous artesunate, three sessions of hemodialysis, three doses of dihydroartemisinin-piperaquine (DHP), and a single dose of primaquine. The patient's condition improved significantly without any sequelae, and he was discharged and evaluated in the outpatient clinic.

## 3. Discussion

**Figure 3:**
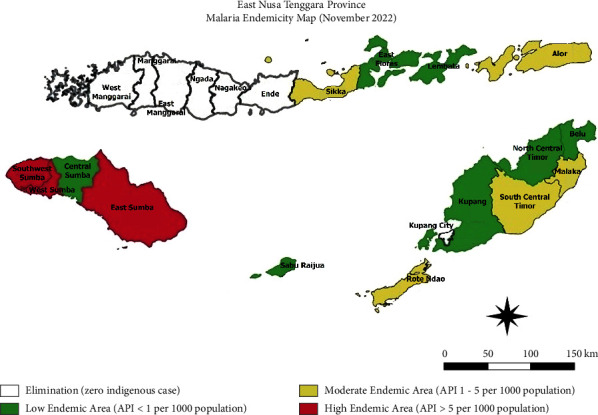
Malaria endemicity map of East Nusa Tenggara province (November 2022).

Malaria continues to be a major global health problem, an epidemic in tropical and subtropical regions of Asia, America, and Africa [3]. Indonesia is one of the malaria-endemic countries, and the majority of malaria cases are concentrated in Eastern Indonesia. The following provinces have the high prevalence of malaria: Papua, West Papua, East Nusa Tenggara, North Maluku, and Maluku [4]. As the Indonesian Ministry of Health has a target to eliminate malaria transmission completely by 2030, in recent years, the malaria elimination program in Indonesia has shown particularly positive progress. Between 2007 and 2017, the annual parasite incidence (API) in Indonesia fell by three times, from 2.89 per 1000 to 0.9 per 1000. [5] According to a local provincial report, in 2022, East Nusa Tenggara province has declared major reduction in malarial cases with an API value of 2,2 per 1000 population. Of 22 districts, there are 12 districts categorized as low-endemic areas (API <1 per 1000), and 7 districts have reached zero transmission [6]. Within East Nusa Tenggara province, the highest prevalence of malaria is in Sumba Island, while more than half of the districts in Flores Island have officially been reported malaria-free. This achievement marked that East Nusa Tenggara province has gradually approached its malaria elimination goal (Figure 3). In this case, the patient came from East Flores district in Flores Island, categorized as a low-endemic area.

Severe malaria is most commonly caused by infection with *Plasmodium falciparum*. The World Health Organization defines specific clinical findings and laboratory results for severe malaria [1]. Cerebral malaria had the highest case fatality rate ranging from 16% to 35%, whereas respiratory distress had a case fatality rate of 20.9% [7]. Another study revealed that the mortality of severe malaria was higher in patients with impaired consciousness (26.5%), severe anemia (11.5%), and acute kidney injury (10%) [8]. Severe manifestations in this case report were impaired consciousness with convulsion, acute kidney injury, jaundice, anemia, and copious hematemesis.

Cerebral malaria is a fatal neurological complication that mostly occurs in children and pregnant women, while the mortality rate of cerebral malaria is higher in adults [9]. In our case, severe malaria likely occurred due to a delay in the diagnosis. In a systematic review and meta-analysis, Mousa et al. showed that a prolonged treatment delay (3-≥4 days) was at a 2.4-fold risk of developing cerebral malaria versus treatment ≤24 hours [10]. The neurological damage induced by cerebral malaria may be lethal when treatment is delayed or in the setting of limited resources [11]. Our patient is a young immunocompetent adult, and he had a *Plasmodium falciparum* parasite density of 3.5%. This parasitemia could have potentially induced endothelial lesions of the blood-brain barrier, resulting in brain ischemia and neurological damage. Fortunately, after intravenous artesunate and supportive treatment were initiated periodically, his consciousness gradually improved, and he was discharged without any neurological deficit.

According to the WHO criteria for severe malaria, AKI is defined as a serum creatinine >3 mg/dL [1]. It is associated with a mortality as high as 75% when renal replacement therapy (RRT) is not started in time [12]. The incidence of AKI in severe malaria patients varies across studies, with the AKI incidence rate reached as high as 52.5% in malarial patients [13–15]. Prompt dialysis has been associated with a 25% mortality reduction and a 30% improvement in renal function among malarial patients with AKI [16]. On admission, our patient showed elevated urea and serum creatinine levels with significant uremic manifestations. Thus, he underwent emergent hemodialysis. During hospitalization, laboratory results showed gradual renal function improvement after three hemodialysis sessions.

The presence of jaundice in malaria indicates a more severe illness with a higher incidence of complications and mortality. Jaundice developed in our patient was caused by hepatic dysfunction and damaged red blood cells during acute malaria illness, resulting in anemia and black water fever [17]. Black water fever is hemoglobinuria from massive intravascular hemolysis, characterized by the black-tea color of urine, as seen in our patient. Black water fever also occurs in G6PD deficiency patients who take particular drugs. However, we cannot perform G6PD examination as it is unavailable in our hospital. Subsequently, after days of treatment, our patient showed improved transaminases and bilirubin levels, and urine color progressively switched from black-tea to yellowish color (Figure 2).

The presence of rapidly progressing anemia in malaria needs prompt treatment [18]. During the acute phase of the illness, our patient showed progressive anemia; thus, he received packed red cell blood transfusion. Interestingly, our patient also showed massive hematemesis when admitted to our hospital. Even though complicated falciparum malaria may develop bleeding manifestations, they rarely present with overt bleeding in adults [19]. The pathogenesis of bleeding in malaria is believed to result from platelet dysfunction and coagulopathy which leads to thrombocytopenia [20]. Albeit gastrointestinal endoscopy was not performed, we suggested that the stress-related gastric erosions in severely ill conditions were the sources and that thrombocytopenia with dysfunctional platelets could have been the etiology of hematemesis in our patient. In studies evaluating gastric-related symptoms in malarial patients, mucosal edema and congestion (gastritis) were the majority endoscopic findings; thus, gastritis should be considered in malaria infection [21, 22].

The risk of mortality is increased if treatment of uncomplicated malaria is delayed. The possibility of mortality increased by almost four times in patients who delayed consultation by a day compared to those who came in very early [23]. Recognizing and prompt treatment is, therefore, of vital importance. The highly variable presentation of malaria may mimic that of many other diseases creating misdiagnosis [1]. This case was initially suspected as dyspepsia syndrome before being referred to our hospital. After two weeks since the initial symptoms appeared, the patient was diagnosed with *Plasmodium falciparum* malaria and developed severe complications. He was treated in the intensive care unit because of multiorgan dysfunction due to the delayed malaria diagnosis. We thought that the delayed diagnosis is due to lack of consideration of malaria as differential diagnosis, even in low-endemic areas, causing severe complications similar to our case. Recent local data showed that East Nusa Tenggara province has declared major reduction of malaria cases and most of the districts have been downgraded to low transmission area [6]. This important achievement, however, may cause inadvertence and reluctance of physician to perform malarial screening. The patient came from a low malaria-endemic district (East Flores) in Flores Island; hence, severe and life-threatening complications should not have occurred if diagnosis and surveillance of malaria had been performed properly. Fortunately, prompt treatment with intravenous artesunate and other appropriate treatment prevented the patient from deteriorating.

Therefore, to modify the mortality risk, even in low-endemic malaria areas, physicians should remain meticulous and have high suspicion of malaria in patients presenting with nonspecific manifestation. Additionally, lack of experience in malaria diagnosis and misdiagnosis of diseases in primary health care lead to delayed diagnosis and life-threatening complications, ultimately increasing the mortality risk among patients [24].

## 4. Conclusion

Negligence and late diagnosis of malaria infection lead to severe complications and a worse prognosis. While several factors may influence a patient's prognosis, intravenous artesunate must be initiated as early as possible to maximize the chance of recovery. Close monitoring and other supportive medical care are also critical. Despite major reduction of malaria cases in endemic areas, physicians should remain meticulous and eager to perform malarial screening even without specific manifestations.

## Figures and Tables

**Table 1:** Patient's laboratory findings during hospitalization.

Parameters	Reference	Day of hospitalization									
		1	2	3	4	5	6	7	9	10	13
Hemoglobin (g/dL)	11.0–17.6	9.8	9.1	7.2	7.0	9.6	10.3	10.3	9.2		
Hematocrit (g/dL)	35.0–55.0	24.7	23.5	18.7	17.8	25.3	27.5	30.8	26.2		
Leukocyte (10^3^/*μ*L)	4.0–12.0	7.97	10.46	12.09	16.65	15.03	12.59	16.5	13.2		
Thrombocyte (10^3^/*μ*L)	150–440	61	70	139	203	222	239	257	262		
Total bilirubin (mg/dL)	0.2–1.0		32.41			18.32				11.46	
Direct bilirubin (mg/dL)	0.1–0.2		21.67			12.64				5	
AST (U/L)	<37	214	91			24					
ALT (U/L)	<42	409	277			64					
Natrium (mmol/L)	136–145	121	123			137					
Potassium (mmol/L)	3.5–5.2	6.1	4.5			4.9					
Chloride (mmol/L)	96/106	93	95			102					
Urea (mg/dL)	17.1–42.8	529	316	429		212	229		98		44
Creatinine (mg/dL)	0.7–1.3	12.8	8.3	12.29		7.79	7.19		2.37		1.16
BUN (mg/dL)	7–21	247	148	199		99	107		46		21
Malaria blood smear	Negative	Positive	Positive	Positive	Negative	Negative	Negative	Negative	Negative		

AST, aspartate aminotransferase; ALT, alanine aminotransferase; BUN, blood urea nitrogen.

## Data Availability

The data used to support the findings this case are included within the article.
